# Circadian rhythms and circadian clock gene homologs of complex alga *Chromera velia*

**DOI:** 10.3389/fpls.2023.1226027

**Published:** 2023-12-08

**Authors:** Jitka Richtová, Olga Bazalová, Aleš Horák, Aleš Tomčala, Vijaya Geetha Gonepogu, Miroslav Oborník, David Doležel

**Affiliations:** ^1^ Biology Centre, Academy of Sciences of the Czech Republic, Institute of Parasitology, České Budějovice, Czechia; ^2^ Biology Centre, Academy of Sciences of the Czech Republic, Institute of Entomology, České Budějovice, Czechia; ^3^ Department of Molecular Biology, Faculty of Science, University of South Bohemia, České Budějovice, Czechia; ^4^ Faculty of Fisheries and Protection of Waters, University of South Bohemia, Vodňany, Czechia

**Keywords:** apicomplexa, *Chromera velia*, circadian clock, cryptochrome, zoospore formation

## Abstract

Most organisms on Earth are affected by periodic changes in their environment. The circadian clock is an endogenous device that synchronizes behavior, physiology, or biochemical processes to an approximately 24-hour cycle, allowing organisms to anticipate the periodic changes of day and night. Although circadian clocks are widespread in organisms, the actual molecular components differ remarkably among the clocks of plants, animals, fungi, and prokaryotes. *Chromera velia* is the closest known photosynthetic relative of apicomplexan parasites. Formation of its motile stage, zoospores, has been described as associated with the light part of the day. We examined the effects on the periodic release of the zoospores under different light conditions and investigated the influence of the spectral composition on zoosporogenesis. We performed a genomic search for homologs of known circadian clock genes. Our results demonstrate the presence of an almost 24-hour free-running cycle of zoosporogenesis. We also identified the blue light spectra as the essential compound for zoosporogenesis. Further, we developed a new and effective method for zoospore separation from the culture and estimated the average motility speed and lifespan of the *C. velia* zoospores. Our genomic search identified six cryptochrome-like genes, two genes possibly related to *Arabidopsis thaliana CCA/LHY*, whereas no homolog of an animal, cyanobacterial, or fungal circadian clock gene was found. Our results suggest that *C. velia* has a functional circadian clock, probably based mainly on a yet undefined mechanism.

## 1 Introduction

Circadian oscillators are part of the cell’s endogenous mechanism for maintaining synchrony with daily environmental changes during the day and night cycle. They have been studied in many multicellular organisms but are also known from unicellular eukaryotes and prokaryotes (Huang et al., 1990). In some model organisms, such as *Arabidopsis thaliana*, *Neurospora crassa*, *Mus musculus*, *Drosophila melanogaster*, and *Synechococcus*, the components and mechanism of circadian clocks are known in great detail (Kondo and Ishiura, 2000; Baker et al., 2012; Mohawk et al., 2012; Nakamichi, 2020; Doležel, 2023), while in others, the system is just being described (Poliner et al., 2019; Farré, 2020; Borges-Pereira et al., 2021).

The simplest circadian mechanism described to date, the post-translationally controlled Kai oscillators, operates in phototrophic prokaryotes and is based on the cooperation of only three proteins: KaiA, B, and C (Johnson et al., 2017; Swan et al., 2018). Studies have shown that the circadian machinery of eukaryotes relies on a similar mechanism of several interlocked transcription-translation feedback loops (TTFL) controlled by environmental factors such as light and temperature (Poliner et al., 2019). Although they operate on the same general principle of TTFL, the key components of the circadian mechanism are substantially different among organisms. The formal rules vary among genetic communities; here we use capital italicized abbreviations for genes (CCA) and capital abbreviations for proteins (CCA). *Neurospora crassa* served as a model organism to identify the fungal circadian clock. Its mechanism is based on the interplay between FRQ (frequency) and WC-1,2 (white-collar) from PAS (PER/ARNT/SIM) protein family (Crosthwaite et al., 1997; Poliner et al., 2019). The PAS family proteins also form the core of the animal circadian clock mechanism represented by PERIOD, and basic helix-loop-helix PAS (bHLH-PAS) proteins CYCLE, CLOCK, and BMAL, whose activity is inhibited by animal-type cryptochromes (Dunlap, 1999; Poliner et al., 2019). Involvement of bHLH-PAS protein RITMO1 was reported for the marine diatom *Pheodactylum tricornutum*, and homologous sequences were discovered also in alveolates, cryptophytes and rhodophytes (Annunziata et al., 2019). However, the bHLH-PAS/RITMO1 protein was found in early branching alevolates, heterotrophic cilliates, and in dinotoms, dinoflagelates that harbour the diatom endosymbiont. It is very likely that the bHLH-PAS protein in dinotoms is present in the diatom endosymbiont, which is almost unreduced and contains a diatom nucleus, mitochondrion, and plastids (Yamada et al, 2017). The clock system of phototrophs is unique for the prevalence of repressor elements (Nohales and Kay, 2016). Contrary to expectations, no homolog of the cyanobacterial Kai family gene was found in eukaryotic phototrophs. The circadian mechanism of green algae and plants is characterized by the presence of MYB family proteins (such as LHY, Late Elongated Hypocotyl and CCA1, Circadian Clock Associated) and pseudo response regulators (PRRs; Poliner et al., 2019; Petersen et al., 2022).

The circadian rhythms of eukaryotic phototrophs with plastids of red lineage are much less explored. However, with current tools for omics analyses, new promising targets for the study of primary red algae are emerging (Miyagishima and Tanaka, 2021; Hirooka et al., 2022). Most studies of red lineage circadian clocks have been conducted on stramenopiles, a lineage whose plastids evolved through complex endosymbioses. Self-sustaining 24-hour rhythms (running under constant conditions) have been described in diatoms (Ragni and D’Alcalà, 2007), brown algae (Phaeophyceae) (Schmid and Dring, 1992; Schmid et al., 1992) and the eustigmatophyte *Nannochloropsis* (Poliner et al., 2019; Farré, 2020). The molecular model has previously been outlined for the marine diatoms *Phaeodactylum tricornutum* (Annunziata et al., 2019; Farré, 2020; Zhang et al., 2022) and *Skeletonema costatum* (Zhang et al., 2022). RITMO1 is the first and so far the only clock component for stramenopiles. It resembles an animal bHLH-PAS (basic Helix-Loop-Helix PAS) clock component, and the coding gene is widely distributed in marine algal genomes, with exception of dinoflagellates (Annunziata et al., 2019). Other proteins with bHLH-single PAS domains have also been found in dinoflagellates, ciliates, and the cryptophyte alga *Guillardia theta* (Farré, 2020). A special chapter in the circadian story of organisms with complex, rhodophyte-derived plastids should belong to dinoflagellates. Their oscillator is based neither on the eukaryotic TTFLs nor on the post-translational prokaryotic Kai system. Studies have revealed proteins with circadian rhythmicity, but with stable mRNA levels (Morse et al., 1989; Milos et al., 1990; Roy et al., 2014). Dinoflagellates have a unique circadian mechanism based mainly on translational level dynamics, with casein kinase 2 possibly playing a crucial role (Roy et al., 2014; Jadhav et al., 2022). Sporozoa, obligate apicomplexan parasites with cryptic complex plastids of red lineage, must cope with incomparable conditions. For example, their circadian rhythms have been found to be synchronized with the host, but also to function independently. The circadian mechanism in sporozoans remains unknown (Rijo-Ferreira and Takahashi, 2020; Rijo-Ferreira et al., 2020).

The metabolism of phototrophs strongly relies on sunlight, which provides the energy for photosynthesis. The circadian clock system of phototrophs is therefore permeated by various photoreceptors that bring information about the quality, intensity, and direction of light (Nohales and Kay, 2016; Lopez et al., 2021). Photoreceptors differ in the wavelength they absorb. Red and far-red light is absorbed by phytochromes, blue light is predominantly perceived by cryptochromes, phototrophins, and Zeitlupe, and finally UV-light is caught by proteins in the UV RESISTANT LOCUS 8 family (Lopez et al., 2021; Petersen et al., 2021). In addition, the function of photoreceptor homologs may differ between plants and algae (Petersen et al., 2021).

Taken together, the core circadian clock components are unique for specific lineages across the tree of life, including the photosynthetic organisms, when cyanobacteria, green lineage phototrophs and red lineage phototrophs built their oscillatory mechanism based on genes from various progenitors. An exception are cryptochromes that seem to be present at least in all lineages of eukaryotic phototrophs (Farré, 2020; Cordoba et al., 2021; Petersen et al., 2021; Jadhav et al., 2022).

Due to its unique position within the alveolates as the closest photosynthetic relative of apicomplexan parasites (such as malaria’s causative agents *Plasmodium*), *Chromera velia* has been intensively studied since its discovery (e.g. Moore et al., 2008; Kořený et al., 2011; Oborník et al., 2011; Janouškovec et al., 2013). During the peculiar life cycle, *C. velia* periodically undergoes the flagellated zoospore stage (Oborník et al., 2016). It has been shown that zoospores peak about six hours after the beginning of the light phase and disappear before its end (Oborník et al., 2011). In this study, we aim to determine whether this periodicity exhibits circadian behavior and identify putative homologs of circadian genes in *C. velia*.

## 2 Materials and methods

### 2.1 *C. velia* cultivation conditions

*C. velia* (CCMP2878) was obtained from NCMA (Provasoli-Guillard National Center for Marine Algae and Microbiota, Maine, USA) and grown in flasks (Sarstedt T-75, Sarstedt AG & Co. KG, Germany) in Guillard F2 medium (Guillard and Ryther, 1962) at 26°C.

**Figure 1:**
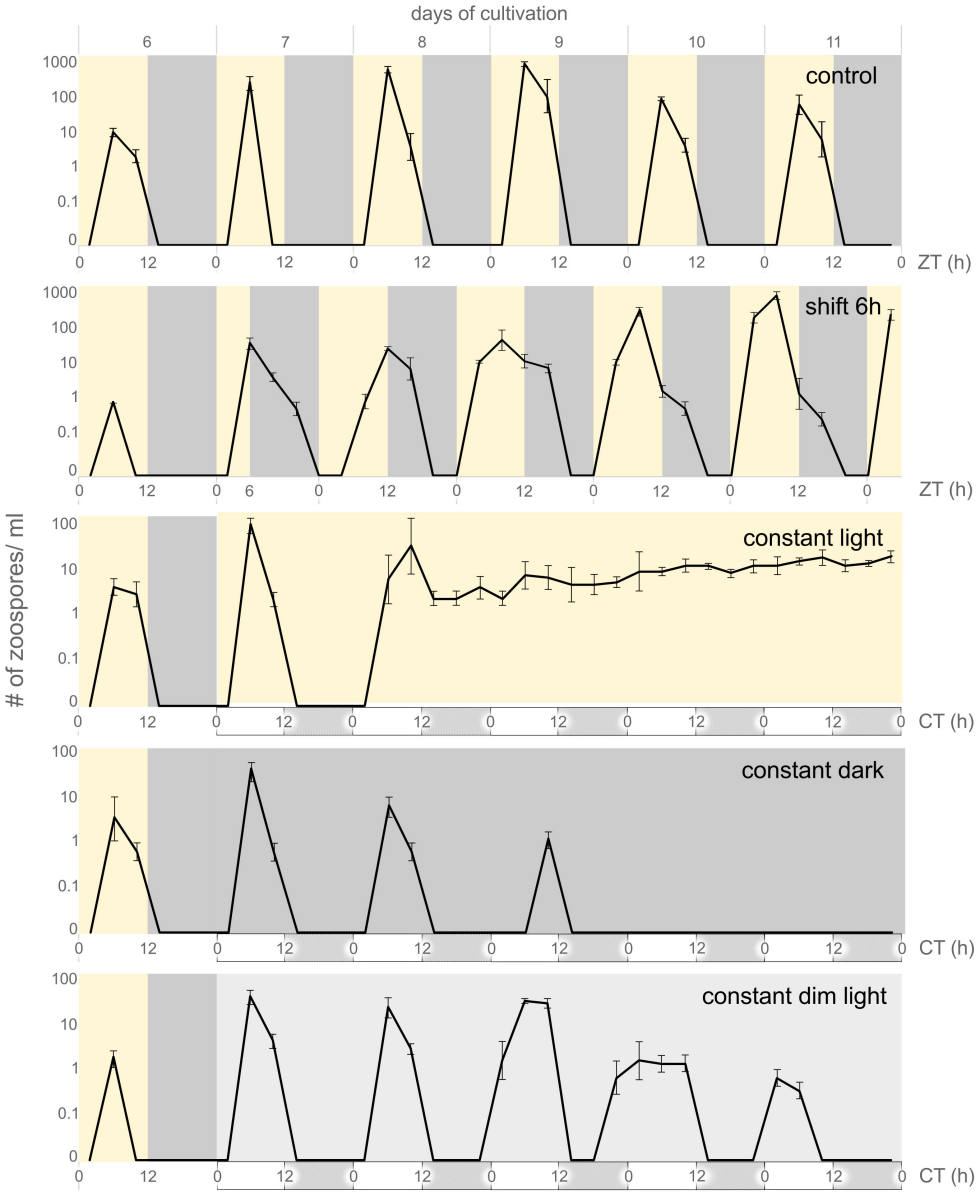
Periodic release of *C. velia* zoospores. Graphs visualize days of active zoosporogenesis (day six to eleven), whole sixteen days of experiment are visualized in **Supplementary Figure 1**. Yellow background indicates light regime, dark grey background indicates dark regime, light grey background indicates constant dim light regime. For constant conditions hypothetical day and night are visualized by white and grey boxes under the timeline, respectively. Zeitgeber time (ZT) or circadian time (CT) is depicted under each regime. Under the 12/12 hours of light-dark regime (control), the release of zoospores occurs in the photophase, with the peak corresponding to ZT 6. If the light period is shifted for six hours towards the dark (shift 6h) the zoosporogenesis continues with the same phase during the day seven and eight of cultivation. The transition phase can be observed within next two days (day nine to eleven) and the rhythmic release is synchronized with the new regime after 120 h (day twelve, see **Supplementary Figure 1**). Culture transition to constant light led to continuous zoosporogenesis starting after 36 hours in constant conditions. The zoosporogenesis in constant dark and constant dim light regimes periodically continued for three or five more cycles, respectively, and then ceased. The zoosporogenesis was renewed when the culture from constant dark condition was placed back to the light/dark regime (see **Supplementary Figure 1**).

For the experiment to quantify the release of zoospores into the culture under different light conditions (control LD, shift +6h, constant light, constant dark, constant dim light; **Figure 1**), three replicates were inoculated and cultivated for sixteen days under the following conditions: 100 ml flasks at 12/12 light/dark regime (LD) under a white light source in the range of 380-720 nm (PowerGlo, Hagen Group®, UK) with an intensity of 100 μmol.m-2s-1. On the second day of zoospore release (seventh day of cultivation) the light conditions were changed as follows: i) control – no change in cultivation conditions; ii) shift +6h – light/dark regime was shifted towards the dark period for six hours and then remained in 12/12 hours light dark/regime, iii) constant light – flasks were cultivated under constant light conditions (100 μmol.m-2s-1) with no dark period in between, iv) constant dark – the flasks were cultivated under constant dark conditions starting at 0h of the seventh day of cultivation and remained in constant dark for the next six days (until the twelfth day of cultivation) when conditions were shifted back to 12/12 hours light/dark regime (100 μmol.m-2s-1), v) constant dim light- flasks were cultivated under the constant dim light of the intensity (30 μmol.m-2s-1) from 0h of the seventh cultivation day.

The effect of light wavelength on zoosporogenesis was observed on cultures grown in 12/12 light/dark regime under LED light sources (OWIM GmbH & Co., Neckarsulm, Germany) with the following characteristics. The wavelength of the red LED light was 628 nm with an illumination intensity of 130 μmol.m-2.s-1. The wavelength of the green LED light was 515 nm with an illumination intensity of 130 μmol.m-2.s-1. The wavelength of the blue LED light was 456 nm with an illumination intensity of 130 μmol.m-2.s-1. The white light source (control, standard cultivation light conditions) was in the range of 380-720 nm (PowerGlo, Hagen Group®, UK), with an illumination intensity of 100 μmol.m-2.s-1. All cultures were observed and cells and zoospores were counted every day at 6h (ZT) for six days in a row.

The intensity of the illumination was measured with the Portable Luxmeter HD 2102.1 (Delta OHM, Padova, Italy). Light spectra were measured with Pasco Spectrometer PS-2600 (Roseville, California, USA).

### 2.2 Counting of *C. velia* cells

Cultures of *C.velia* were checked and cell numbers were counted every four hours (ZT=2, 6, 10, 14, 18, 22) using an inverted microscope (Motic, San Antonio, Texas, USA). Culture density (cells/ml) and the number of zoospores (zsp/ml) were manually counted in the Bürker counting chamber under 200x magnification.

### 2.3 *C. velia* zoospore separation

A transparent 50 cm long glass U-tube with an inner diameter of 0.8 cm was filled with 20 ml of F2 medium and placed in the stand. Half of the U-tube was wrapped with aluminum foil. Four ml of *C. velia* culture with active zoospores were added to the unwrapped part of the U-tube. The glass U-tube was placed under the white light source in the range of 380-720 nm (PowerGlo, Hagen Group®, UK), with an illumination intensity of 100 μmol.m-2.s-1. After 40 minutes, the aluminum foil was removed and 2 ml of the upper layer of the F2 medium containing concentrated zoospores was separated into a clean tube and the zoospores were observed under the microscope.

To count the speed of *C. velia* zoospores the culture with active zoospores (400 zoospores/ml) was loaded in the glass U-tube as described above. 50 µl of F2 medium were transferred from the aluminium-covered part of the U-tube to the wells of the 96 well plate every minute, starting from the first minute after *C. velia* culture insertion. This was repeated 36 times (till the concentration of zoospores/ml in the withdrawal was similar to the number of zoospores in the loaded sample, i.e. approximately 20 zoospores in 50 µl). The wells were then examined for the presence of zoospores, and their number in the field of view was counted under 100x magnification. The experiment was performed in three replicates.

To estimate the average lifespan of zoospores, zoospores were separated using the above-mentioned method. 50 µl of F2 medium containing concentrated zoospores were transferred to a well of a 96-well plate with a rounded bottom in three replicates. Zoospores were observed every 30 minutes. The number of active zoospores compared to newly transformed coccoids was counted until no active zoospores were seen in the well (=100% coccoids in the well). The average lifespan was estimated.

### 2.4 RNA isolation

For RNA isolation and expression analysis, 1500 ml of F2 medium was inoculated with *C. velia* and cultured for 2 weeks in a 12/12 LD regime with a light intensity of 100 μmol.m-2.s-1 at 26°C. Thereafter, one-third of the culture was placed in constant darkness, one-third in constant light (100 μmol.m-2.s-1), and one-third remained in the LD cycle and served as a control. Three replicates were cultivated under each experimental condition. 15 ml samples were collected at given time points during the first day of each regime, centrifuged (3820 g for 10 min at 4°C), and the cell pellet was stored at 80°C. All manipulations up to cell lysis were performed under dim red light. Total RNA was isolated using the RNeasy® Plant Mini Kit 50 (QUIAGEN), residual genomic DNA was removed using the RNAse free DNA set (QUIAGEN), and the efficiency of this depletion was confirmed by PCR.

### 2.5 Gene expression analysis

Quantitative PCR (qPCR) was used to determine gene expression (Kobelková et al., 2010). 1 µg of RNA was used for cDNA synthesis with Super Script ® III reverse transcriptase (Invitrogen), random DNA hexanucleotides (Generi Biotech) and nuclease inhibitor RNAsin (Promega). cDNA was diluted 15× for qPCR. Each reaction contained 3μl of diluted cDNA, 25 pmol of both primers (listed in **Supplementary Table 1**), 6 μl IQTM SYBR® Green Supermix 2x (Bio-Rad), and 2.5 μl ddH2O. Amplification was run in C1000 Thermal Cycler (Bio-Rad): initial denaturation for 3 min at 95°C, followed by 10 sec at 95°C, 20 sec at 50°C, 20 sec at 72°C, 40 cycles. Final melting analysis from 65°C to 95°C served as a control for product size. Primer sequences used for qPCR are listed in **Supplementary Table 1**. All data were normalized to the relative levels of the gene encoding ribosomal protein 49 (*rp49*). To minimize pipetting errors, all reactions were performed in triplicate.

### 2.6 Circadian clock gene identification and phylogenetic analyses

The genome sequence and predicted proteins of *C. velia* and *V. brassicaformis* available in CryptoDB (https://cryptodb.org/cryptodb/app) were analyzed using two parallel approaches. In the first steps of the study, we used the protein BLAST algorithm and searched for plant-specific (APRR5/7/9, TOC, ZTL, CCA1, CHE, GI, TOC, ZTL, CCA1, phytochrome from *Arabidopsis thaliana*, cryptochrome), animal-specific (TIM, PER, PDP1, CLK, CYC, VRI from *Drosophila melanogaster*, TAI from *Pyrrhocoris apterus*; Smýkal et al., 2023), Cryptochrome-related insect Photolyase (Bazalová and Doležel, 2017), light-insensitive insect Cryptochrome (Bajgar et al., 2013) fungal-specific *(*white collar 1, WC1, white collar 2, WC2, frequency from *Neurospora crassa*), algal-specific (*RITMO1* and *bHLH1b*), and bacterial-specific (phytochrome, kaiA-C from *Synechococcus elongatus*) circadian gene homologs and important photoreceptors. All query proteins used are listed in **Table 1**. Identified hits were manually evaluated whether the similarity encompasses variable or conserved (functionally important) regions of the proteins. Reciprocal BLAST searches were performed in *Arabidopsis*, animals, fungi and bacteria. The identified sequences were aligned using MAFFT, and FAST tree algorithm (both in Geneious software, Geneious) were used for quick phylogenetic analyses to clarify, whether the identified hits branch in the target protein group or not. Reasonable candidates were used for more detailed phylogenetic analysis (see below).

In the second phase of the study, we repeated the search for circadian clock homologs using HMMER3. For each reference gene, we first identified and extracted 250 ‘best homologs’ (according to the blastp MaxScore) from NCBI nr database. After filtering out sequences with more than 99% similarity, these datasets were aligned using the local pair algorithm as implemented in MAFFT (Katoh, 2002). From these alignments, we have created HMMER profiles using the *hmmbuild* tool from HMMER 3 (hmmer.org). Profiles were then searched against predicted *C. velia* and *V. brassicaformis* predicted proteins using the *hmmsearch* (HMMER 3 package) with evalue threshold set to 1e-15.

Top hits were examined for the presence of key protein domains and/or compared with the complete sequence of the query circadian clock protein. In addition, for each putative homolog of reference circadian genes, we have constructed phylogenetic dataset comprising of sequences from reference hmmer profile, *C. velia* and *V. brassicaformis* homologs and other putative homologs from custom protein database covering known eukaryotic and prokaryotic diversity. The datasets were aligned using the local-pair algorithm implemented in MAFFT. Ambiguously aligned regions were removed using TrimAl (Capella-Gutiérrez et al., 2009) with -gappyout option invoked. For each dataset, maximum likelihood phylogeny was inferred using IQTree2 (Minh et al., 2020) under gamma-corrected LG model. All the phylogenetic trees were inspected. Those, where the topology suggested possibility that the *C. velia* and/or *V. brassicaformis* sequences are homologues of given reference circadian gene, we visually inspected the sampling, removed eventual redundancy, realigned the dataset and repeated maximum likelihood inference, this time with the best -fitting model selected based on Bayesian Information Criterion score as estimated by ModelFinder implemented in IQTree2 (Kalyaanamoorthy et al., 2017). For CPF dataset, the LG+C40+F+R9, for *cca1*/*lhy* dataset the LG+C40+R5, and for *ztl* dataset the LG+C20+F+R7 were used. Non-parametric bootstrap was inferred from 1000 replicated using the ultra-fast algorithm (-bb 1000 setting) also in IQTree2.

*C. velia* homologs of the cryptochrome-photolyase family were screened for the presence of conserved motifs using InterPro (Blum et al., 2021). The presence of an N-terminal targeting signal was checked using ASAFind (Gruber et al., 2015; Armenteros et al., 2019). The presence of nuclear localization signal (NLS) was checked using NucPred (Brameier et al., 2007), cNLS Mapper (Kosugi et al., 2009), NLStradamus (Nguyen Ba et al., 2009), and INSP (Guo et al., 2020). Only those parts of the sequence were considered positive for NLS where at least two predictors matched.

## 3 Results

### 3.1 Rhythmicity of *C. velia* zoosporogenesis under different light conditions

Previous studies on the life cycle of *C. velia* have shown that zoospores are periodically released into culture and that this event is closely associated with the light phase. It has also been show that zoospore abundance reaches its maximum at ZT 6 (Oborník et al., 2011). We monitored the zoospore abundance in the culture inoculated to fresh medium (1:1000, approximately 2 cells/ml) for over two weeks under different light conditions (see Materials and Methods for details). Under constant 12/12 light-dark conditions (control), zoospores were first detected on day six and reached a maximum on day nine. In the control, zoospores were periodically present for ten consecutive days, reaching a maximum at ZT 6 each day; no zoospores were observed on the day sixteen (**Supplementary File 1**, control).

When the culture conditions were changed (seventh day of cultivation) by shifting the light/dark phase by six hours toward the night phase, the zoospore release into the culture followed the original light/dark cycle for 24 hours, with zoospore abundance peaking at ZT 12 (corresponding to ZT 6 of the original regime). After 48 hours, we observed the prolonged peak of zoospore abundance in the culture, which included both the original and the shifted regimes. After 72 hours, zoospore release was fully adjusted to a new shifted light regime (**Figure 1**, shift 6h) which persisted until the end of the experiment (**Supplementary File 1**, shift 6h).

Constant light conditions resulted in continuous zoosporogenesis that began 30 hours after the transition from the standard regime to constant illumination. Continuous zoosporogenesis lasted for another eight days, but, we observed a lower number of zoospores compared to the previous two cycles (**Figure 1**; **Supplementary Figure 1**, constant light). When the cultures were transferred from standard conditions (12/12 light-dark) to a constant dark regime (seventh day), zoosporogenesis continued for three more cycles under entrained conditions. On the tenth day, no more zoospores were observed in the culture (**Figure 1**, constant dark). Zoosporogenesis resumed when cultures were transferred back to standard conditions (thirteenth day of cultivation; **Supplementary File 1**, constant dark). Cultures transferred to constant dim light conditions led to the longest retention of entrained light/dark cycle, resulting in five more zoosporogenesis cycles after conditions were changed (**Figure 1**, constant dim light). No zoospores were observed in the constant dim light conditions on the twelfth day of cultivation and thereafter.

### 3.2 The influence of light spectra on zoosporogenesis and zoospores

**Figure 2:**
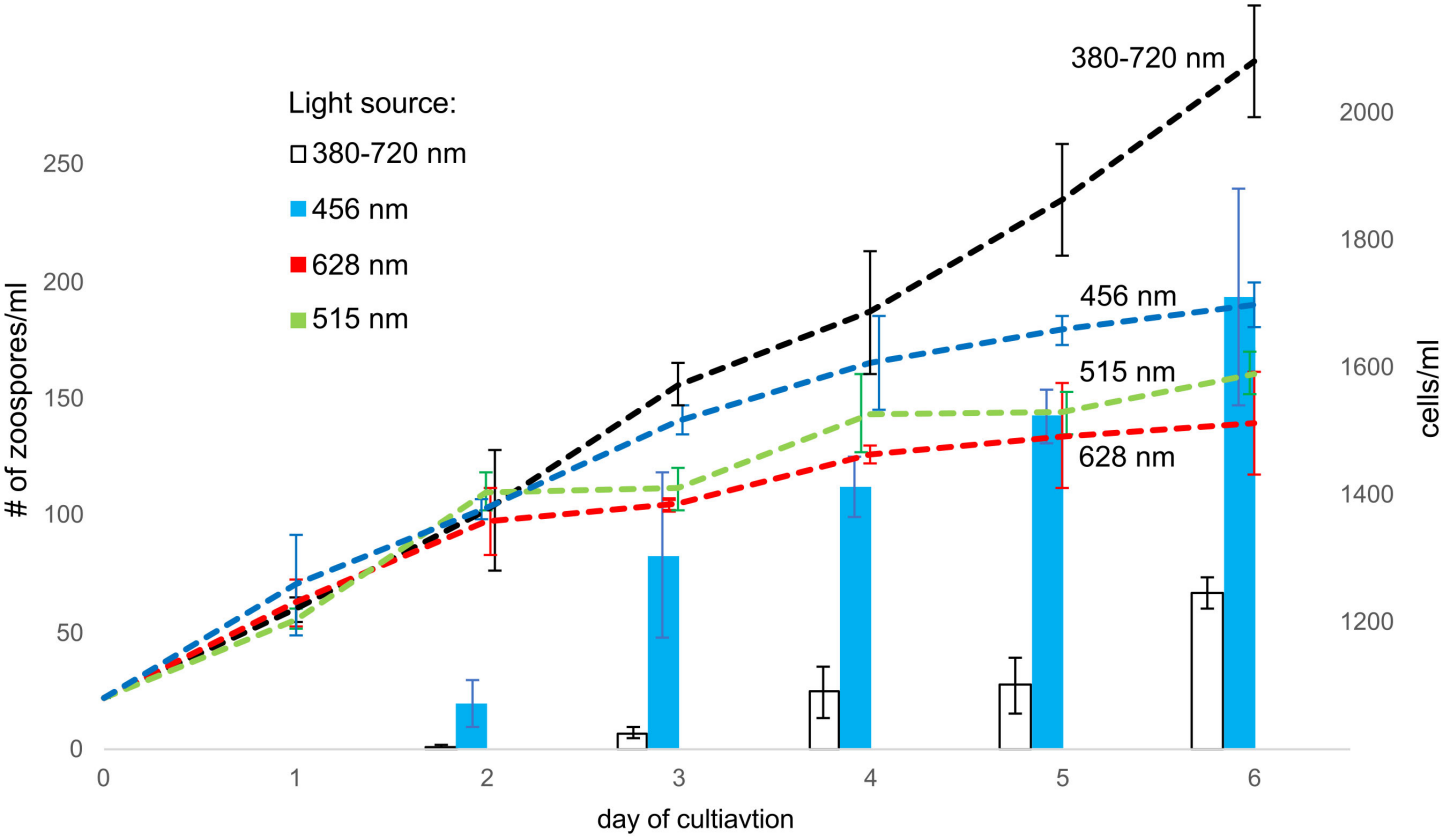
The effect of various light spectra to zoosporogenesis. The columns indicate the number of zoospores in each milliliter of medium (left y-axis), whereas the number of all cells is indicated by dashed lines (right y-axis). Zoosporogenesis is induced by white and blue light. White light (380-720 nm) is the most efficient for *C. velia* growth, but zoosporogenesis is three-fold higher under the blue light (456 nm). In contrast, no zoospores are detected under the green (515 nm) and the red (628 nm) light.

The circadian rhythms of phototrophs are mainly controlled by the light received by photoreceptors (Petersen et al., 2021). Because these photoreceptors absorb different wavelengths, we cultivated *C. velia* under four different spectral conditions: blue, green, and red-light LED and standard white light as a control (see Materials and Methods for details). The control culture, cultivated with white light, grew gradually, and zoospores were observed as early as the second day of cultivation, appearing regularly on the following days. Cultures cultivated under a single wavelength grew more slowly but steadily compared to the control culture. The most striking difference was in zoosporogenesis, which was observed only under blue light and not under green and red light (**Figure 2**). Moreover the culture grown with blue light showed at least three times higher abundance of zoospores than the control culture.

With the exception of red light, *C. velia* zoospores exhibit photophobicity to all light spectra tested, as evidenced by rapid movement away from the light source. The average speed of *C. velia* zoospores was estimated to be about 2 cm/min. We used this light avoidance response to develop a method for isolating pure zoospores and found that the average lifespan of the zoospore is more than 7 hours (see Materials and Methods for details and **Supplementary Figure 3**).

### 3.3 Identification and phylogenetic analysis of circadian clock genes homologs

We first examined the genome of *C. velia* (Woo et al., 2015) for homologs of known core circadian clock component genes from cyanobacteria, fungi, animals, plants, *Chlamydomonas*, and diatoms. Blast search identified putative homologs of cryptochrome photolyase (CPF) protein family and the *A. thaliana* circadian clock genes *CCA1/LHY*. Using the more sensitive approach employing HMMER package, we were able to expand the set of candidates of circadian components in *C. velia* also for *APRR5*/*7*, *CHE*, *GI*, *TOC1*, and *ZTL* from plant circadian system of *A*. *thaliana*. We also identified putative homologs of animal-specific *tim* from *Drosophila melanogaster*, fungal-specific *(wc1*/2*)* and bacterial phytochrome. With the exception of CPF, CCA1/LHY and ZTL, detailed phylogenetic inspection of all candidate protein sequences revealed that most of these candidates branched way out of the reference clades and are thus most-likely false positives.

**Figure 3:**
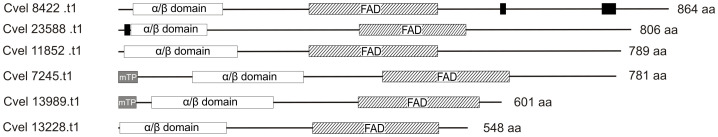
Conserved protein domains encoded by identified homologs of the cryptochrome-photolyase family. The alpha/beta photolyase domain and flavin adenine dinucleotide (FAD)- binding domain form the typical photolyase homology region. mTP, mitochondrial transit peptide. Nuclear localization signals are indicated by black boxes.

Considering the striking effect of blue light on the life cycle of *C. velia*, we decided to investigate the CPF homologs in more detail. We analyzed the amino acid sequence of CPFs for the presence of conserved motifs and organellar targeting signals (see Materials and Methods for details). All *C. velia* CPF homologs possess the typical photolyase homology region (PHR), a conserved sequence at the cryptochrome amino terminus consisting of the alpha/beta photolyase domain and the FAD domain (**Figure 3**). The N-terminal alpha/beta domain of *C. velia* CPF homologs exhibits sequence similarity with the MTHF and HDF chromophore, although Cvel 8422.t1 shows the highest aa divergence from the conserved aa motifs. Two *C. velia* CPF homologs (Cvel 7245.t1, Cvel 13989.t1) contain recognizable mitochondrial transit peptides (mTP, **Figure 3**). Cvel 8422.t1, Cvel 23588.t1, Cvel 11852.t1, and Cvel 7245.t1 have recognizable C-terminal extension, of 363, 259, 288, and 168 aa respectively, downstream of the conserved FAD domain. In Cvel 13989.t1 and Cvel 13228.t1, there is no such expansion, but only 35 and 45 aa downstream of the FAD domain, respectively (**Figure 3**). Our search for nuclear localization signals (NLS) confirmed their presence in Cvel 8422.t1, where two possible NLS motifs were detected at its C-terminus, and Cvel 23588.t1 where one possible NLS motif was found at the N-terminus upstream of the alpha/beta domain.

To investigate the phylogenetic position of *C. velia* CPFs, a dataset was created containing 6-4 photolyases, CPD photolyases, and all known classes of cryptochromes (Kottke et al., 2017). Although the full topology of the tree was not fully supported, relevant groups appeared in the tree (**Figure S2**): (*i*) CRY-DASH1 group, (*ii*) CRY-DASH/PHR2 group, (*iii*) CRY-DASH2 group, (*iv*) plant CRYs and plant-like CRYs, (*v*) group consisting of 6-4 photolyases and animal CRYs, and (*vi*) class II CPD photolyases.

**Figure 4:**
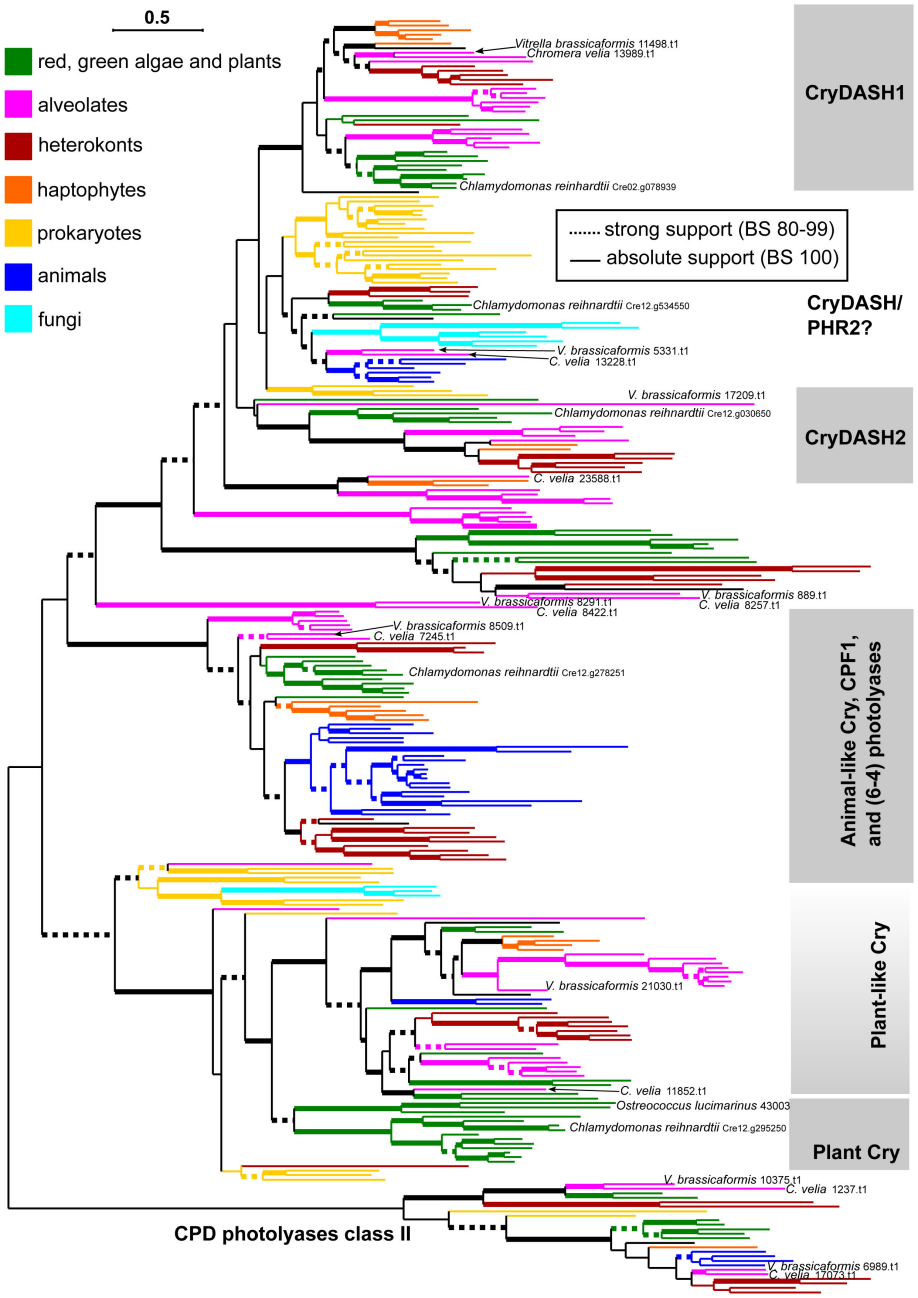
Phylogenetic analysis of cryptochrome photolyase family members from *C. velia*. Color codes for specific eukaryotic and prokaryotic lineages. Terminology for CRY and photolyase groups follows Kottke et al. (2017). For the sake of readability, only *C. velia*, *Vitrella brassicaformis*, and *Chlamydomonas* are highlighted. The fully described tree is in **Supplementary Figure S2**. Tree was inferred using IQTree2 under the LG+C40+F+R9 model, which was found to be the best-fitting according to the Bayesian Information Criterion calculated by model evaluating tool implemented in IQTree2. Non-parametric bootstrap was inferred from 1000 replicated using the ultra-fast algorithm (-bb 1000 setting) also in IQTree2. Thick branches highlight high (dashed) or absolute (full) bootstrap support. The final alignment with ambiguously aligned and gap-rich sites removed using ‘-gappyout’ setting in TrimAl comprised 283 taxa and 534 amino acid positions.

In seven cases (Cvel 13989.t1, Cvel 13228.t1, Cvel 8257.t1 Cvel 8422.t1, Cvel 7245.t1, Cvel1237.t1, and Cvel 17073.t1), CPFs of *C. velia* branched together with the paralog from *Vitrella brassicaformis* (**Figure 4**; **Supplementary Figure S2**), whereas Cvel 23588.t1 and Cvel 11852.t1 occurred only in *C. velia*.

The Cvel 13989.t1 CPFs homolog of *C. velia* clustered within the CryDASH1 group: as the sister taxon of *V. brassicaformis*, haptophytes, and plants. Within CryDASH/PHR2, Cvel 13228.t1 and *V. brassicaformis* branched alongside sequences from prokaryotes, fungi, and animals. Also of interest is the position of Cvel 8422.t1 and *V. brassicaformis* 8291.t1, which form a distinct lineage at the base of all CryDASH tree groups (CryDASH1, CryDASH2, and CryDASH/PHR2). The relationship of *C. velia* to a plant ancestor was confirmed by the position of two CPFs: Cvel 11852 branches within plant-like Cry, and Cvel 7245.t1 branches (together with *V. brassicaformis*) at the base of 6-4 photolyases near plants, diatoms, and heterokonts (**Figure 4**; **Supplementary Figure S2**).

**Figure 5:**
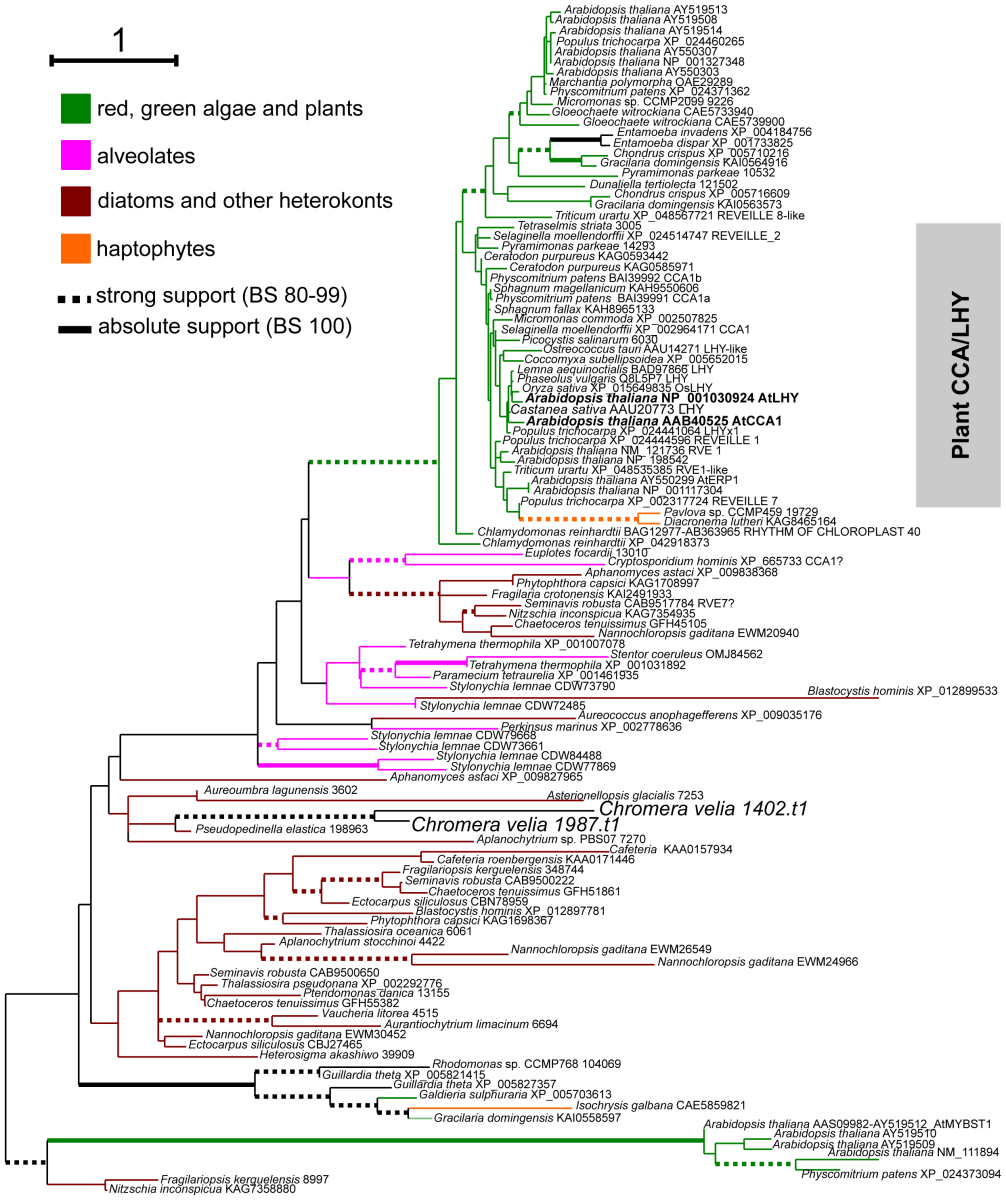
Phylogenetic position of LHY-MYB homologues identified in *C. velia*. The tree was inferred in IQTree2 under the LG+C40+R5 model found to be the best-fitting according to the Bayesian Information Criterion calculated by model evaluating tool implemented in IQTree2. Non-parametric bootstrap was inferred from 1000 replicated using the ultra-fast algorithm (-bb 1000 setting) also in IQTree2. Thick branches highlight high (dashed) or absolute (full) bootstrap support. The final alignment with ambiguously aligned and gap-rich sites removed using ‘-gappyout’ setting in TrimAl comprised 116 taxa and 65 conserved amino acid positions.

The search for the *cca1* and *lhy* homolog yielded two candidates, Cvel 1402.t1 and Cvel 1987.t1. The part of the proteins that we could unambiguously align was too short (40 aa) to draw accurate conclusions about its origin. Nevertheless, phylogenetic analysis positioned both proteins as sister taxa at the base of a clade containing several Myb-proteins, including the circadian components CCA1 and LHY from *Arabidopsis thaliana* and their homologs from green plants and the moss *Physcomitrium patens* (**Figure 5**).

### 3.4 Expression patterns of circadian genes homologs in LD/LL/DD regime

**Figure 6:**
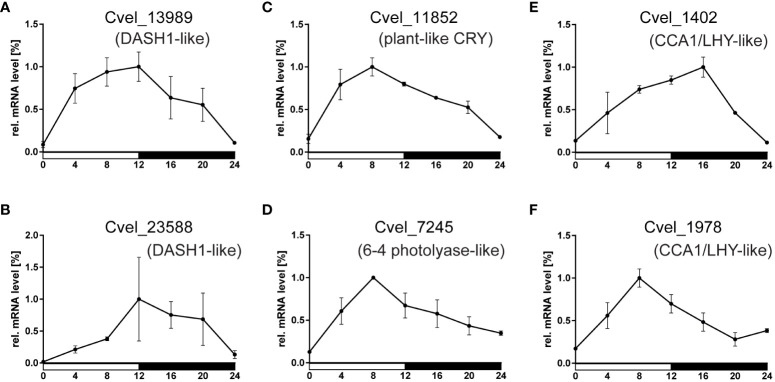
Relative mRNA levels of gene expression in (*C*) *velia* during the light-dark cycle. The expression levels of **(A, B)** two *cry* genes of the DASH1-like group, **(C)** plant-like type, **(D)** 6-4 photolyase type, and **(E, F)** two homologs of CCA/LHY1 were quantified using the expression of the housekeeping gene *rp49* as a reference. The white horizontal bar underneath x-axis corresponds to photophase (light on), and the black bar indicates scotophase (dark). Note that although all six measured transcripts are cyclically expressed, their maxima are at different timepoints.

To elucidate the possible role of circadian homologs found in *C. velia*, we applied quantitative PCR and analyzed the expression patterns of four *cryptochrome* and both *CCA1/LHY* homologs at seven time points during the LD cycle. Remarkably, all six transcripts examined showed clear cyclic expression, albeit with peaks at different time points (**Figure 6**). Importantly, the phase differed between the transcripts examined: Both DASH1-like homologs (*Cvel_13989* and *Cvel_23588*) peaked at the end of photophase at ZT12, while the plant-like (*Cvel_11852*) and 6-4 photolyase-like transcripts (*Cvel_7245*) peaked at ZT8, whereas the greatest difference in expression of *CCA1/LHY*-like transcripts was observed, when *Cvel_1402* peaked at ZT16, whereas *Cvel_1978* peaked 8 hours earlier at ZT8.

Our repeated attempts to quantify cyclic expression during the first two days in DD failed because of low expression of all genes tested. During LD cycles, the mean cycle threshold (ct) in quantitative PCR was ~26 cycles for the reference transcript *RP49*, whereas in constant darkness, the ct of *RP49* dropped to 32-36 cycles. A comparable decrease of ~6-8 cycles was observed for *Cvel_1978* transcripts, and for cry homologs. This overall low expression prevented any meaningful quantification of the qRT PCR data.

## 4 Discussion

Our planet revolves around the Sun and rotates around its own axis (Copernicus, 1543). This facts, which causes an alternation of day and night, has influenced all life forms that have ever lived on Earth (Giebultowicz, 2004). The phenomenon of circadian rhythms was first described in 1729 by Jean-Jacques d’Ortous de Mairan using the periodical movement of Mimosa leaves. Circadian rhythms are still being studied and are not fully understood, especially in non-model organisms. Recently, circadian rhythmicity has even been found in unicellular endoparasites (Rijo-Ferreira and Takahashi, 2020).

*Chromera velia* is a free-living marine alga attracting attention from the scientific community because it is the closest known photosynthetic relative of obligate sporozoan parasites such as *Toxoplasma* and *Plasmodium* (Moore et al., 2008). The life cycle of *C. velia* involves the process of zoospore formation, which is controlled by light and occurs with a periodicity of 24 hours (Oborník et al., 2011). In this work, we 1) investigated the influence of light on zoospore formation of *C. velia*, 2) searched for the presence of known circadian homologs and 3) described their phylogeny, 4) examined the amino acid sequence of CPF homologs for the presence of conserved motifs and target sequences, 5) developed a method for isolation of pure *C. velia* zoospores from the culture and 6) measured the rhythmicity of the mRNA level for some of the homologs found.

Although photosynthetic organisms have many physiological processes that are influenced by circadian rhythms, not all of them can be easily followed *in vivo* (Farré, 2020; Jadhav et al., 2022). Zoosporogenesis of *C. velia* can be easily observed with an ordinary microscope under appropriate conditions. The release of zoospores occurs rhythmically, beginning at dawn, peaking at midday, and declining before the dusk (Oborník et al., 2011). The zoospore release under constant dark continues only for three cycles. In contrast, zoospore release remains robust under constant light, but is non-cyclical. When a *C. velia* culture maintained in a standard 12/12 light/dark regime is transferred to constant dim light conditions, the rhythmic release of zoospores can be followed for five more cycles during the subjective day. This periodic release of zoospores under constant conditions suggests the presence of a ‘ticking’ circadian clock in *C. velia*. The existence of this internal timing mechanism is also confirmed by our phase shift experiments, in which *C. velia* did not respond immediately to the new light regime. Instead, the release of zoospores continued in a similar phase for another cycle, and the broader distribution of zoospores lasted even longer, eventually leading to a successful phase shift. Indeed, the ability to adjust to a new regime, in contrast to an immediate response, is the hallmark of circadian clocks (Roenneberg and Morse, 1993).

From a practical point of view, the more cycles under constant conditions, the better the experimental possibilities. The transition to constant light led to disruption of the cycle, leading to continuous zoosporogenesis from the second day of the transition (**Figure 1**), indicating a high light sensitivity of this process. Determining the exact reactivity spectrum of *C. velia* zoosporogenesis to light intensity would be informative to some extent, but it is beyond the scope of this manuscript.

The role of *C. velia* zoospores is likely to be to disperse in the environment in search of sufficient living conditions and the zoospores are supposed to be an infective stage. In contrast to the second chromerid *V. brassicaformis* (Füssy et al., 2017), no fusion of zoospores was observed and the key meiotic genes are also absent (Vazač et al., 2018). *C. velia* infects both the ectoderm and endoderm of coral larvae (Cumbo et al., 2013; Mohamed et al., 2018). Zoospores are best able to carry out the infection process because they possess the conoid (Oborník et al., 2011; Portman et al., 2014), the conserved core element of apical complex that enables the invasion process in Sporozoa (Kořený et al., 2021). Both activities, opening up a new environment and searching for a host, require a rapid movement that is energetically demanding. The long lifespan of *C. velia* zoospores may require photosynthetic activity even in the motile stage of the alga, and photosynthesis may also support the proposed photoparasitic lifestyle (Oborník, 2020). With increasing depth, the composition of the light spectrum changes rapidly and red light is almost absent below 15 m. At depths greater than 30 m, blue light becomes increasingly enriched (Duanmu et al., 2017). Here we have shown that for successful transition from coccoids to zoospores, the blue spectrum of the light source is a necessary condition (**Figure 2**). Blue-light-driven release of zoospores could be an efficient way to maintain this important cell transition process at greater depth. We observed that zoospores are photophobic. Therefore, *C. velia* zoospores most likely actively search for an environment with lower radiation intensity. By colonizing a shaded environment, *C. velia* expands its ecological niche where it can thrive thanks to its adapted photosynthetic apparatus (Kotabová et al., 2014). It was found that zoospores are able to actively invade the coral larvae (planula, Cumbo et al., 2013). Planulae form new coral colonies by dropping to the seafloor (Dana, 1846). Therefore, pointing the light source in the opposite direction of the zoospore may be helpful in finding a new host. Since we know the average speed and lifespan of the *C. velia* zoospore (see chapter 3.2), we can assume that the zoospore can swim over eight meters in search of a suitable environment after its release from the zoosporangium.

We searched the currently available genome of *C. velia* (Woo et al., 2015) for homologs of known blue light receptors and were successful only for cryptochromes. The only light receptors present in most evolutionary lineages (Lopez et al., 2021) that evolved from photolyases, blue light-dependent DNA repair enzymes (Ahmad and Cashmore, 1993; Hegemann, 2008; Petersen et al., 2021). In *C. velia* we found six homologs of cryptochrome photolyase family proteins (**Table 1**). Our phylogenetic analyses show that the *C. velia* homologs of the CPF family are scattered throughout the tree and have a representative in plant-like, animal, and CRY-DASH1,2 families (**Figure 4**). Cvel_8422.t1 is located at the base of the CRY-DASH group along with the *V. brassicaformis* homolog. Some CRY-DASH proteins retain DNA repair activity under stringent conditions (Chaves et al., 2011) and may reside in organelles (Kleine et al., 2003; Rredhi et al., 2021). Nuclear localization signal has been confirmed at the C terminus of mammalian cryptochromes (Mei and Dvornyk, 2015). Localization of cryptochromes in plants can vary between the nucleus and cytosol and is light-dependent (Xu et al., 2009). We screened CPF homologs of *C. velia* for the presence of conserved motifs and localization signals. In all homologs, we found both alpha/beta photolyase and FAD domains that typically form a photolyase homology region located at the N-terminus of the cryptochrome, suggesting an active role in the light-mediated response and a possible role in the mechanism of *C. velia* circadian clock (**Figure 3**.; Petersen et al., 2021).

**Table 1:** Summary of putative clock gene homologs identified via genome mining in *C. velia*.

	Protein domain(s)	protein name, acc. #, (organism) used for the BLAST	candidate found in *C. velia*
PLANTS	Myb	circadian clock associated 1 (CCA1), P92973.1 (*Arabidopsis thaliana*)	Cvel_1402 Cvel_1978
		late elongated hypocothy (LHY), Q6R0H1.2 (A. *thaliana*)	
		Reveille 8, Q8RWU3.1 (A. *thaliana*)	
		Lux At3g46640 (A. *thaliana*)	
		timing of CAB expression (TOC1), Q9LKL2.1 (A. *thaliana*)	–
		Pseudo-response regulator 3 (PRR3), Q9LVG4.1 (A. *thaliana*)	
		PRR5, Q6LA42.2, (A. *thaliana*)	
		PRR7, Q93WK5.1, (A. *thaliana*)	
		PRR9, Q8L500.2, (A. *thaliana*)	
	PAS, F-box, KELCH	Zeitlupe (ZTL), Q94BT6.2, (A. *thaliana*)	Cvel 15907 Cvel 8260 Cvel 15509 Cvel 4661 Cvel 18659 Cvel 23748 Cvel 23236 Cvel 8482 Cvel 22468
		GIGANTEA, Q9SQI2.2, (A. *thaliana*)	–
	Zinc finger	CONSTANS, Q39057.1, (A. *thaliana*)	–
	PAS, GAF	phytochrome B, P14713.1, (*A. thaliana*)	–
	Cryptochrome / photolyase	cryptochrome 1, Q43125.2, (A. *thaliana*)	Cvel_13989 Cvel_7245 Cvel_11852 Cvel_23588 Cvel 8422 Cvel 13228 Cvel 8257 Cvel 1237 Cvel 17073
		cryptochrome 3, 2J4D_B, (A. *thaliana*)	
ANIMALS		cryptochrome1, AAF55649.1, (*Drosophila. melanogaster*)	
		cryptochrome 2, AGI17567.1, (*Pyrrhocoris apterus*),	
		Photolyase, XM_011293895.2, (*Musca domestica*)	
		Timeless, AAN10371.3, (*D. melanogaster*)	–
	PAS	Period, P07663.2, (*D. melanogaster*)	–
	bHLH-PAS	Circadian Locomotor Output Cycles Kaput (CLOCK), AAC53200.1, (*D. melanogaster*)	–
		cycle, NP_524168.2, (*D. melanogaster*)	–
		brain-muscle-ARNT-like protein 2a (BMAL), AAF88141.1, (*Mus musculus*)	–
		Steroid receptor coactivator TAIMAN (*P. apterus*)	–
	bZIP	Vrille, AHN54139.1, (*D. melanogaster*)	–
		Par domain protein 1 (PDP1), AAF04509.1, (*D. melanogaster*)	–
FUNGI	PAS	frequency (FRQ), P19970.3, (*Neurospora crassa*)	–
		white collar 1 protein (WC1), ESA41980.1, (*N. crassa*)	–
		white collar 2 (WC-2), XP_963819.2, (*N. crassa*)	–
	LOV	vivid, 2PD7_B, (*N. crassa*)	–
CYANOBACTERIA		KaiA, Q79PF6.1, (*Synechococcus elongatus*)	–
		KaiB, Q79PF5.1, (*S. elongatus*)	–
		KaiC, Q79PF4.1, (*S. elongatus*)	–
ALGAE	bZIP, LOV	aureochrome 1-5, CBJ25875.1, CBN74167.1, CBN77970.1, CBJ30909.1, CBJ30584.1, (*Ectocarpus siliculosus*)	–
	bHLH-PAS	RITMO1 (*Phaeodactylum tricornutum*) bHLH1b (*P. tricornutum*	–
		RNA-binding protein C3, EDP06114.1, (*Chlamydomonas reinhardtii*)	–
		RHYTHM OF CHLOROPLAST 15, BAG12976.1, (*C. reinhardtii*)	–
		RHYTHM OF CHLOROPLAST 40, BAG12977.1, (*C. reinhardtii*)	–
		RHYTHM OF CHLOROPLAST 55, BAG12978.1, (*C. reinhardtii*)	–
		RHYTHM OF CHLOROPLAST 66, BAG12979.1, (*C. reinhardtii*)	–
		RHYTHM OF CHLOROPLAST 75, BAG12981.1, (*C. reinhardtii*)	–
		RHYTHM OF CHLOROPLAST 114, BAG12982.1, (*C. reinhardtii*)	–

The C-terminus of cryptochromes is much more variable but has a major impact on cryptochrome function and is often involved in interactions with partner proteins (Chaves et al., 2011). The longest C-terminus is located in Cvel_8422.t1, where the two NLSs were also detected, suggesting that this CPF homolog can be targeted to the nucleus (**Figure 3**). The potential NLS domain was also predicted at the N-terminus of the Cvel_23588.t1, the CPF homologue with second longest aa sequence. The potential DNA repair activity of these homologs is difficult to verify because there is no expression system in *C. velia*. Organellar targeting signals were detected for the animal-like CPF homolog (Cvel_7245.t1) and one of the two CRY_DASH homologs (Cvel_13989.t1), both of which have a mitochondrial transit peptide (**Figure 3**). None of the examined *C. velia* CPF homologs had a positive plastid targeting signal. Mitochondrial targeting of the above- mentioned CPF homologs may enhance the physiological response of *C. velia* to environmental changes, thereby increasing its flexibility.

In *Arabidopsis*, two closely related myb proteins expressed in dawn, CCA1 and LHY, are required for clock function. Their homologs are likely found in all green plants, including the moss *Physcomitrium* patens (Okada et al., 2009; Cervela-Cardona et al., 2021). Our BLAST search revealed only two candidate myb-like proteins in *C. velia*. Although phylogenetic analysis was relatively limited due to the short and divergent sequence, both *C. velia* homologs branched at the base of a large clade containing CCA1 and LHY from the green plastid lineage. However, several other myb-containing proteins from *A. thaliana* are part of this clade. So far, there is no functioning transformation system in *C. velia*. Thus, without functional evidence, the role of the myb homologs from *C. velia* in the clock remains unclear.

A characteristic feature of circadian rhythms is their rhythmic oscillation within a 24-hour period. This rhythmicity is entrained by external cues (such as a light source) but persists under constant conditions within a nearly 24-h period even in the absence of external stimuli (Kuhlman et al., 2018; Farré, 2020). An easily detectable physiological feature with circadian properties in *C. velia* is zoosporogenesis (**Figure 1**). Currently, three main types of circadian oscillators are known: eukaryotic systems relying on interlocked transcription-translation feedback loops (TTFL; Lande-Diner et al., 2013), prokaryotic post-translationally controlled Kai oscillators (PTO; Golden and Canales, 2003), and presumably ancient metabolic/redox oscillators conserved in all lineages (Edgar et al., 2012).Within the eukaryotic TTFL system, we can also identify the type of circadian clock conserved in bilaterian animals (Kotwica-Rolinska et al., 2022), with some components present even in basal animal lineages such as cnidarians (Reitzel et al., 2010). The plant system relies on different proteins involved in TTFLs that are conserved, with some idiosyncrasies, from moss to Arabidopsis (Holm et al., 2010). The existence of another oscillator cannot be ruled out, since dinoflagellates do not fit into either the TTFL or PTO oscillators and the metabolic/redox oscillator function has not been proven in them (Jadhav et al., 2022). The circadian cycle of most dinoflagellate proteins has been found to be regulated at the translational level (Milos et al., 1990; Roy et al., 2014; Jadhav et al., 2022).

Although the bHLH-PAS protein has been found in ciliates, an early branching alveolates (Annunziata et al., 2019), it is probably absent in advanced alveolates such as dinoflagellates, apicomonads (including *C. velia*), and sporozoans. Dinotoms, dinoflagellates with the diatom endosymbiont are probably the only exception, but the bHLH-PAS/RITMO1 protein is probably present in the unreduced diatom endosymbiont rather than in the dinoflagellate host cell. Therefore, it appears that the particular type of circadian clock that uses bHLH-PAS/RITMO1 proteins has been lost with the acquisition of the complex plastid in alveolates (dinoflagellates, apicomonads, and sporozoans).

*C. velia*, together with other Apicomplexa, forms a sister branch of dinoflagellates (Moore et al., 2008) and inhabits a biogenic sediment of stony corals (Mathur et al., 2018), periodically releasing zoospores into the environment (Oborník et al., 2011). We developed a new method for separating the zoospores of *C. velia* that will certainly be helpful in experimental work with this life stage, which is probably involved in the infection of coral larvae. Our observations showed that the periodicity of zoosporogenesis follows almost a 24-hour free-running rhythm. In our search for homologs of circadian clock genes, we found two genes possibly related to *A. thaliana CCA/LHY*. We also found six homologs of blue light photoreceptors from the cryptochrome family. However, without functional transformation tools in *C. velia*, we cannot demonstrate their role in the circadian rhythm of *C. velia*. Thus, although the circadian clock exists in *C. velia*, its molecular mechanism remains elusive and could either rely on clock gene homologs that are so modified that they could not be identified, or the *C. velia* clock uses completely different clock components.

## Data availability statement

The original contributions presented in the study are included in the article/**Supplementary Materials**, further inquiries can be directed to the corresponding authors.

## Author contributions

JR prepared **Figures 1**-**3** and **Supplementary Figure 1**. AH prepared **Figures 4**, **5** and **Supplementary Figure 2**. DD prepared **Figure 6**, **Table 1**, and **Supplementary Table 1**. JR and DD designed and analyzed experiments for circadian rhythmicity and the influence of light wavelength. JR and VGG performed the time laps counting. AT run the cultivations for expression analyses. OB and DD designed and analyzed the gene expression part of the research. AH and DD identified homologs and reconstructed their phylogeny. DD and MO designed and led the study. JR, DD, AH, and MO wrote the paper, with input from all authors. All authors contributed to the article and approved the submitted version.
