# Supplementary figures and images for: Circadian rhythms and circadian clock gene homologs of complex alga Chromera velia
Source: Front Plant Sci. 2023 Dec 8;14:1226027. doi: 10.3389/fpls.2023.1226027 (PMC10739334; doi:10.3389/fpls.2023.1226027)

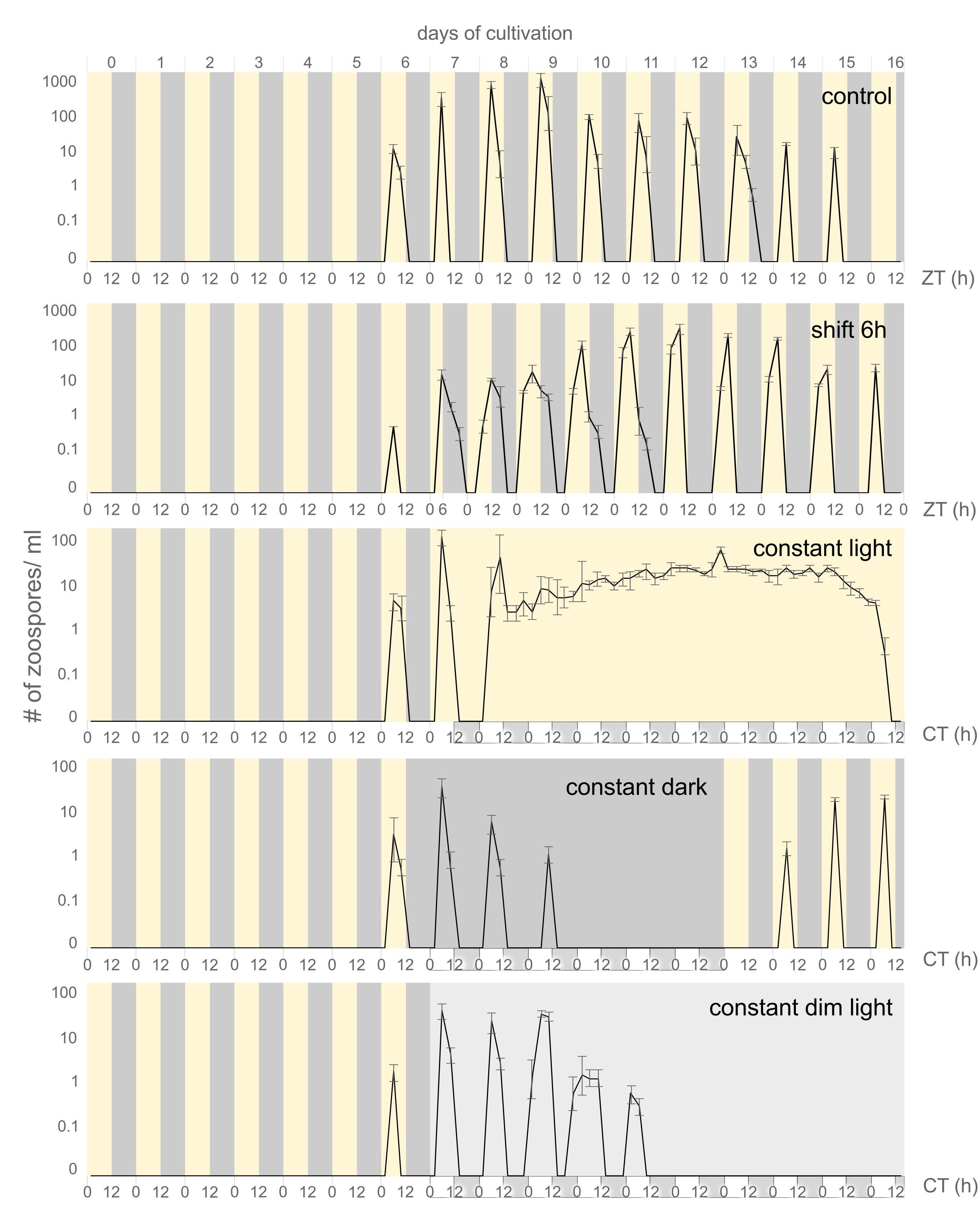

Supplement: Supplementary file 1 [file Image_1.tif]

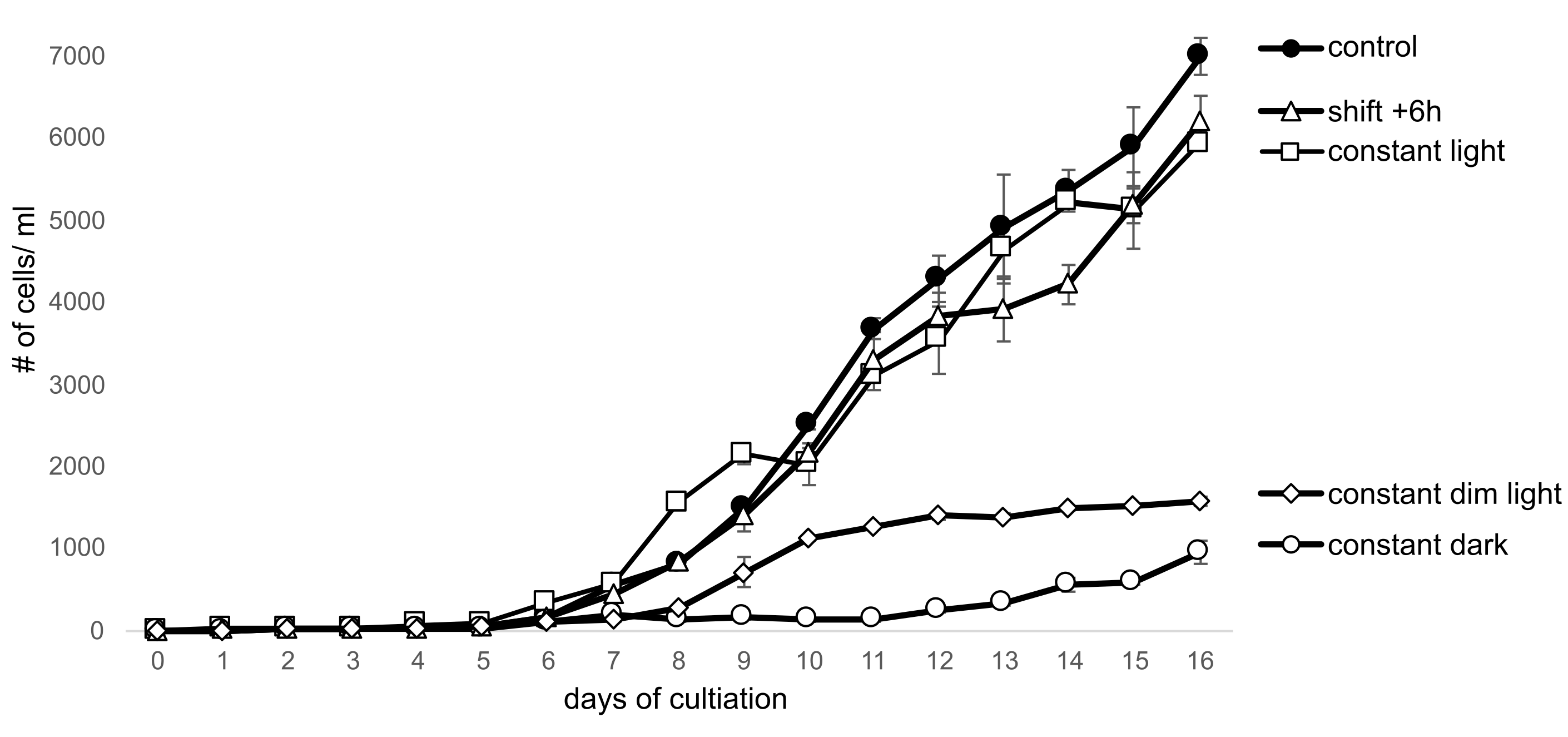

Supplement: Supplementary file 2 [file Image_2.tif]

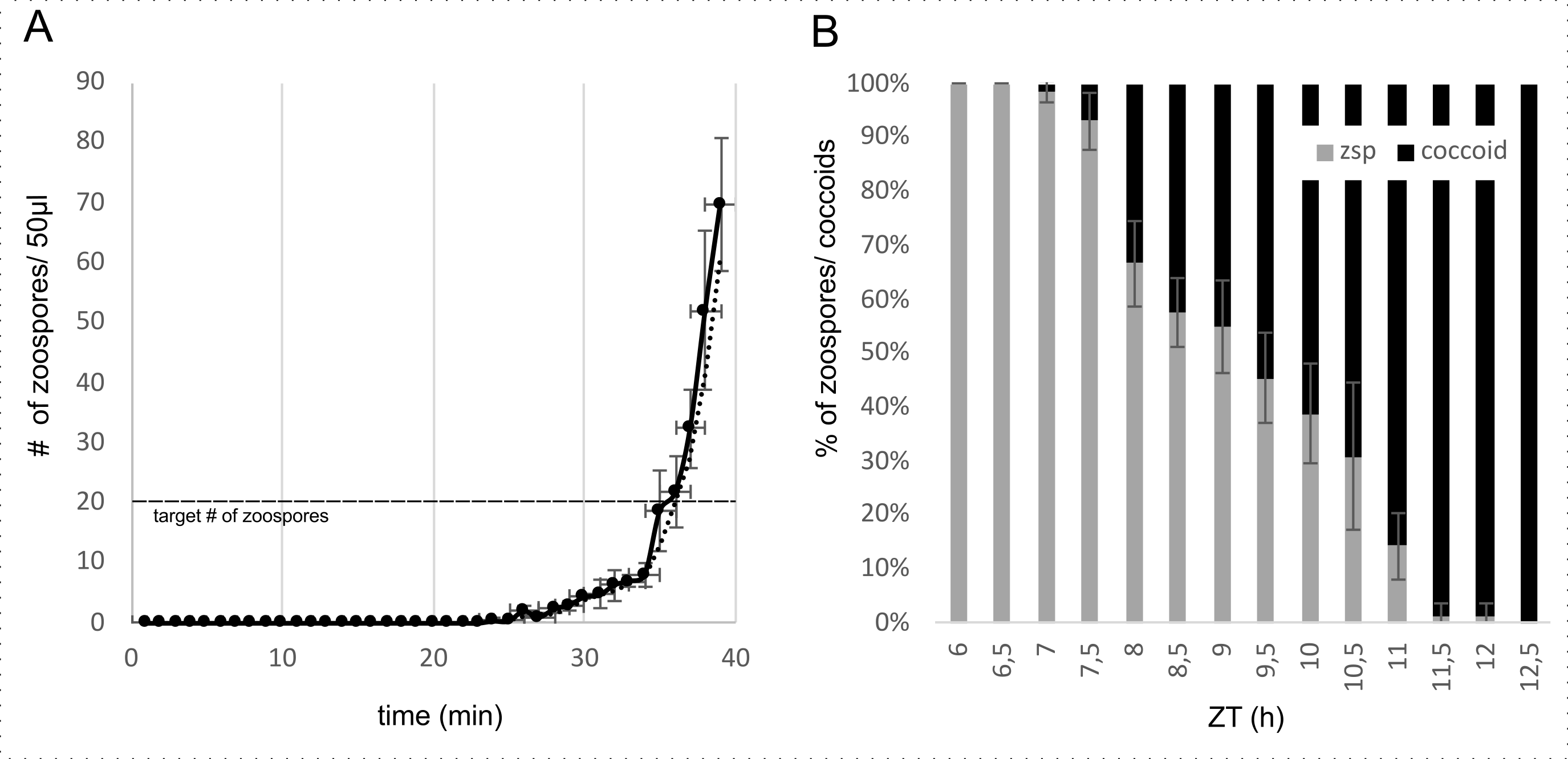

Supplement: Supplementary file 3 [file Image_3.tif]

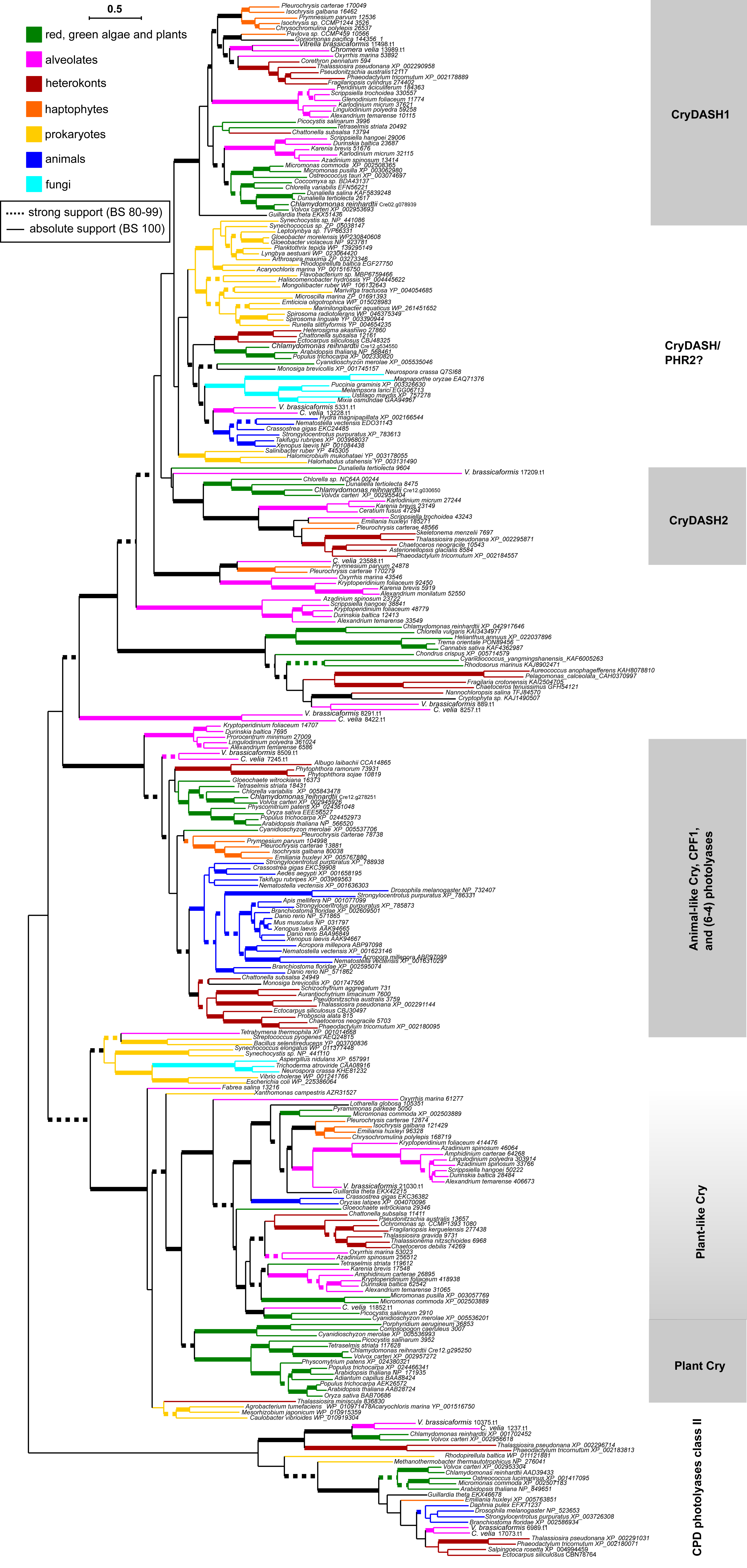

Supplement: Supplementary file 4 [file Image_4.tif]

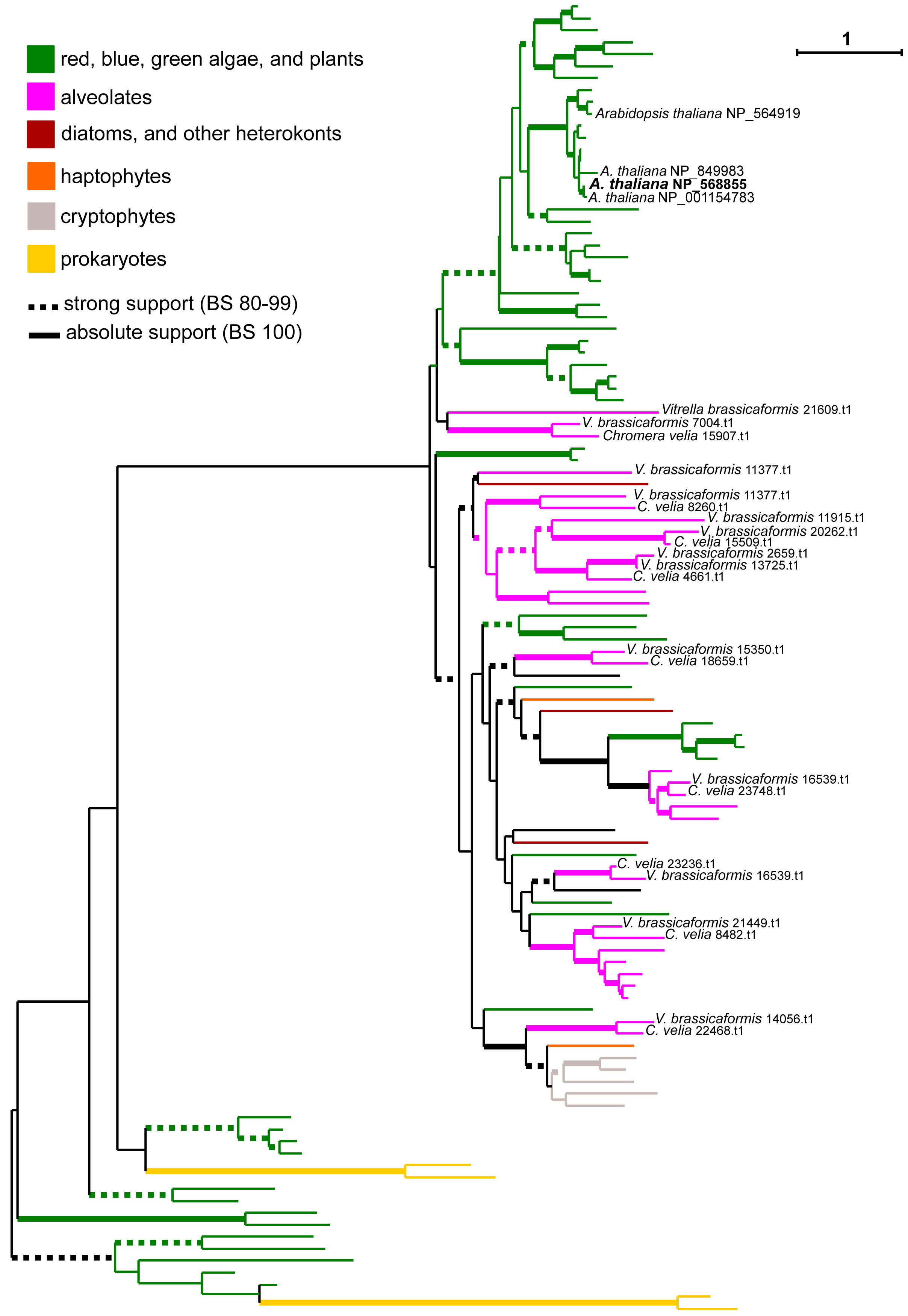

Supplement: Supplementary file 5 [file Image_5.tif]
