# Supplementary material for: Circadian rhythms and circadian clock gene homologs of complex alga Chromera velia
Source: Front Plant Sci. 2023 Dec 8;14:1226027. doi: 10.3389/fpls.2023.1226027 (PMC10739334; doi:10.3389/fpls.2023.1226027)
Supplement: Supplementary file 6 [file Table_1.docx]

Table S1 – primers used in qRT PCR

| **gene** | **forward primer (5´-3´)** | **reverse primers (5´-3´)** |
| --- | --- | --- |
| *Cvel_13989* | GGATTGGGTCGGATGAAGAG | GCGAACTTGGATGAGTAGTCAG |
| *Cvel_7245* | GTCTGCGTCCTGTTTCTTCTACCA | CGTCACTTCCTCACTGTGCT |
| *Cvel_11852* | GCAGTTTGTACGTTCGTGGG | CTCCTCCTCCAGGTCCCTC |
| *Cvel_23588* | GAGTGCTCAGGATGGTTTCT | GCACCTGTAGACCCAATTGA |
| *Cvel_1402* | CCCTCCTCTCGTCCAGTTAG | CGGAGGAATGAGGAGGAAAC |
| *Cvel_1978* | ACAGTCCGTCCGCAGTCGTCTCG | GGTGCCCACATCCCCTCATCGTT |
| *Cv rp 49* | CTATAAGCGTGTCAAGAAGAGC | GAGCATGTCAAGATCCTGGGTGTT |
